# Decision support framework for IT project manager recruitment

**DOI:** 10.1016/j.heliyon.2024.e24685

**Published:** 2024-01-24

**Authors:** Christin Karrenbauer, Jana Gerlach, Michael H. Breitner

**Affiliations:** Leibniz Universität Hannover, Königsworther Platz 1, 30167, Hannover, Germany

**Keywords:** Recruitment, IT project manager, Requirements and benefits, Taxonomy, Archetypes, Decision support framework

## Abstract

Information technology project managers (IT PM) have a critical influence on IT project success while attracting and selecting the right IT PM is challenging. We followed a four-level research design and firstly developed a taxonomy as an input for a cluster analysis to identify patterns in IT PM job advertisements. Based hereon, we developed a decision support framework for IT PM recruitment. We evaluated our findings in an online survey. We identified multiple design elements for IT PM job advertisements within five perspectives and deduced five IT PM archetypes. The decision support framework uses five questions to assist IT PM recruitment. We expand the knowledge base and consider not only IT PM requirements but also benefits. Our decision support framework is the first to holistically support IT PM recruitment, supports recruitment managers in structuring job interviews, identifies potential matches between applicants and recruiters, and assists in the final selection.

## 1 Introduction

This research targets to support decision-making for IT PM recruitment in providing an easy to understand and use framework were recruiters can find the best-suited applicant according to their skills and experiences. The results of the framework, the decision tree, leads to a specific type of IT PM, were a recruiter can check and validate the job advertisement or the candidate during the application process. Many organizations use advanced technologies to react to businesses' changing demands and processes, meet organizational objectives, and remain competitive [1]. Thus, expenditures in information technology (IT) increased in the last years and continue to grow [2]. Nowadays, IT and IT projects have a large share of the total organizational budget [3,4]. However, organizations are often challenged with the successful implementation of IT projects [1] that are characterized by high failure rates [5]. Failure is often caused by organizational, process, stakeholder, and team factors. Especially competent [6], skilled [7], and experienced [8] (project managers with appropriate assignments and leadership have a decisive influence on the project's success [9]. An IT project manager's (IT PM) responsibility is to accomplish project goals by managing the IT project and, for example, identify requirements, establish objectives, and balance time, scope, and budget. Literature emphasizes that successful project management depends on the PM's skills [10]. Thus, various studies have identified the required competencies for an (IT) PM [e.g., [[11], [12], [13]]]. In general, they need different soft- and hard skills as well as business- and technology-specific knowledge [1]. Some studies state that soft skills strongly influence IT project success [e.g., [13]].

The demand for qualified IT project managers has increased and the attraction and selection of the “right” one is a critical business activity and challenge for many organizations [14]. Many positions remain vacant because of this high demand and qualification-related requirements [15]. An adequate selection indicates a high maturity in organizational project management [16]. Although some research compares requirements from literature with requirements extracted from job advertisements, often only a PM and not an IT PM perspective is considered [14,17,18]. Further, past research already focuses on selecting the “right” (IT) PM based on previous performance [e.g. Ref. [19]] and analyzed skills an (IT) PM should have [e.g., [[11], [12], [20]]]. However, different skills lead to different success profiles depending on the conditions for success and the requirements are position dependent and can vary according to the IT PM's tasks [21]. Recruitment is a crucial process for an organization's existence and various factors have an impact. Applicants analyze job advertisements for information including benefits, working conditions, and work environment [22]. Research shows that job advertisements with more information are perceived as more attractive and tend to increase the fit between the organization and the applicant [23]. Thus, it is important to precisely describe the requirements and benefits in an IT PM job advertisement to receive the most suitable applications for the vacant position [24]. Therefore, and along with [24], IT PM job advertisements need to be formulated as precisely as possible to get the attention of the most suitable candidates for an IT PM position. To get this attention from candidates and select the “right” one, practitioners must be supported in IT PM recruitment. However, research is limited in providing a supportive framework for practitioners and academics. There is a lack of research on recruiting IT PM applicants from outside an organization and support the recruitment lifecycle, from writing job advertisements to conducting interviews and making selections. Therefore, we address the following research questions (RQ).

RQ1 What are theoretically grounded and empirically validated elements of IT PM positions and what archetypes can be deduced with this classification?

RQ2 How can a decision tree be designed to support IT PM recruitment?

First, we classify literature and real-world job advertisements for IT PM and develop a taxonomy [25,26]. It enables further theory-building, for example, design theories towards an improved understanding of IT PM job advertisements with required skills and corresponding benefits [25,27]. Following that and to evaluate our taxonomy's applicability, we perform a cluster analysis to identify meaningful archetypical patterns [25]. Identified archetypes are different patterns of job advertisements with different skill requirements and benefit offers. Thus, the clustering techniques go beyond our taxonomy's descriptive character and strengthen its understandability grouping objectives instead of an individual consideration. In contrast to existing literature, we do not only identify important competencies for an IT PM [e.g., [11]]. Together with other skills and corresponding benefits, we use our classification as input for an archetype analysis to identify different patterns in job advertisements. These serve as input for our decision tree, a decision support framework for IT PM job advertisements, interviews, and selection. It enables to define the requirements of an IT PM position using vocabulary that we have defined and then group them into clusters (archetypes). Based on the archetypes, requirements for an IT PM position can be identified. The decision tree then supports writing targeted job advertisements building on the requirements. In addition, the decision tree offers a checklist for the further application process, supports conducting interviews, and can be used as a tool for comparing, selecting, and clustering applicants. Fourth, we evaluate our taxonomy, archetypes, and decision-support framework with seven experts. Overall, our artifacts can support the entire recruitment process, from writing job advertisements to conducting interviews and assisting in selecting IT PMs. In doing so, we first provide the theoretical background before describing our research design and methods. Afterwards, we derive and evaluate our taxonomy, archetypes, and decision-support framework. Finally, we discuss our results and findings, deduce implications and recommendations, and describe limitations, an outlook for further research, and conclusions.

## 2 Theoretical background

### 2.1 IT project manager competencies

IT projects are complex, cross-functional, dynamic, non-routine, temporary, and uncertain [28,29]. This makes them difficult to manage and an IT PM faces several challenges [9,30,31]. At the same time, IT PM have a decisive influence on an IT project's successful completion or failure [32]. Therefore, recruiting the “optimal” IT PM is a critical business activity. It requires someone with various competencies, qualifications, and personality traits [33] to guide the project team through the challenging, stressful, and dynamic IT project environment [34]. Competencies combine an individual's knowledge, skills, attitudes, personal characteristics, and behavior for a defined task [35,36]. According to Bloom's Taxonomy of the Cognitive Domain [37], competencies can be further differentiated into soft skills and hard skills [38]. Soft skills encompass behaviors, traits, and attitudes that enable IT PM to guide, motivate, and influence stakeholders and their project teams. Hard skills are baseline resources needed to perform a task and include, e.g., certificates or technical skills [39]. Because of digitization and an increasingly complex organizational environment, hard and soft skills are indispensable for IT PM to manage their tasks successfully, and these skills increase to their extent [11,12]. Further, project management institutions have developed competency guidelines, such as the Project Management Competence Development Framework [10] and the International Project Management Association Competence Baseline [11,40]. A further particular feature of IT projects is that their managers must have both business-specific knowledge and technology-specific knowledge (hybrid IT PM). The former includes knowledge about business requirements, functionalities, processes, and workflows. Technology-specific knowledge includes, for example, methods and tool competencies and modeling techniques that support IT project execution. They are often referred to as being developed by training and experience. However, rapidly changing and evolving environments and practices make it difficult for IT PM to keep their knowledge up to date [1,41].

Much research has been conducted to identify and analyze the essential competencies of an IT PM. Based on a questionnaire by Refs. [42,43] applied a qualitative survey with 118 participants to identify and rank crucial behavioral skills. They ranked interviewing, directing, and managing as the most required. This was followed by competencies related to communications (speaking, listening, writing) and interpersonal skills (cooperation, patience, sensitivity, diplomacy), with abilities of selling, assertiveness, and nonverbal communication as ranked the lowest. Further, through a repertory grid approach with 19 IT PM [18], identified 46 IT PM skills and grouped them into nine skill categories: client management, communication, general management, leadership, personal integrity, planning and control, problem-solving, systems development, and team development. Based on different combinations of the skill categories, they were further able to build four IT PM archetypes, including general manager, problem solver, client representative, and balanced manager. In a further study based on two iterations and a total of 33 interviews with 21 senior management, technical, and supervisory employees [44], identified soft competencies that IT PM require for successful project completion and rated them according to their importance. They found that skills depend on the specific project phase. In the initiation phase, e.g., effective questioning, feedback generation, and listening skills are critical, while project management skills and knowledge and consensus-building are important skills for the planning phase. Getting along and being results-oriented are crucial for the implementation phase and writing skills for the closeout phase [45]. conducted a qualitative study with ten IT PM. They identified three important issues: the ability to manage multiple modes of communication, the frequency of communication, and the knowledge of when to involve higher authorities to enable cooperation. Further, they found that inexperienced PM are mostly as likely to have competencies as more experienced colleagues.

### 2.2 Related literature on IT PM job analyses

In general, the recruitment process aims to fill vacant positions with employees who have suitable qualifications [22]. [14] examined the recruitment of project managers based on job advertisements and addressed how companies describe the necessary competencies of project managers. In their results, the categories education level, certification, health and safety, knowledge of project management tools, and compliance with regulations are the most common descriptions companies use in their job advertisements for project managers. Drawing upon a Delphi study with 19 IT PM [20], identified 19 skills essential for an IT PM and ranked them in a first iteration. Building on that, they performed follow-up interviews in a second iteration. According to that, leadership, verbal communication, scope management, listening, and project planning skills are crucial. Despite the findings, some research indicates that executives and senior managers focus more on soft skills than hard skills in applications [41]. This is also supported by the findings of a qualitative study with 16 IT PM by Ref. [13]. They found that technological skills were less regarded for IT project success than behavioral, business, and managerial competencies [11]. investigated essential competencies to influence a project's success and analyzed correlations between them. They grouped the competencies into seven groups (leadership, self-management, interpersonal, communication, technical, productivity, and managerial) and identified communication, commitment, and leadership as the most important. According to Ref. [12], PM face new technical and contextual conditions that exceed the previously identified competencies. Thus, they analyzed skills that are required in the context of Industry 4.0. They found that behavioral and soft skills increase in importance while management and technical skills are the most challenging. Further, changed circumstances impact required knowledge, how to communicate and interact, and the work's velocity and capacity [46]. used a literature review and quantitative study to analyze a PM's behavioral skills (leadership, communication, result orientation, emotional intelligence, ethics, creativity and motivation) influence on the success of Information System (IS) projects. Their results significantly impact emotional intelligence, creativity, and ethics having the most influence. In addition to academic research on IT PM, there are various certificates a PM can receive in practice. They confirm that a PM has dealt with certain topics and has at least a baseline knowledge of IT project management methods and tools. However, it does not necessarily mean they are more efficient in managing IT projects. For many IT PM positions, certificates are becoming increasingly important for companies as a hiring criterion [39,47]. Despite the important competencies of an IT PM, it is important to highlight the right competencies and corresponding benefits in job advertisements and to consider them when interviewing and making a final decision on a candidate. A meaningful support for recruitment managers to write the most suitable job advertisement and advise them during the job interview and selection is thus beneficial and value-adding.

## 3 Research design and methods

To address our RQs, we developed and followed a four-level research design. We first (level 1) created a taxonomy of elements for IT PM positions following the taxonomy development method proposed by Refs. [25,26]. Taxonomy development is a commonly used method in Information Systems literature [48] and find application in various areas, e.g., chatbots [49] and business models [50]. In the second level and based on our taxonomy, we performed a cluster analysis to identify archetypes of IT PM positions in line with the recommendations proposed by Ref. [51]. According to Ref. [25], the archetype analysis allows the direct evaluation and approval of the taxonomic results by grouping the classified objects into groups with similar characteristics. In the third level, we developed a decision support framework in the form of a decision tree. As [ [52], p. 7] highlighted “Decision trees can be visualized as tree-like structures, which makes it easy to understand the model's decision-making process. The advantage of decision trees is that they are easy to interpret and understand, making them a good choice […]”. The decision tree is based on our taxonomy and archetype analysis results. According to Refs. [53,54] the evaluation of the results is an essential element in IS research. Therefore, we finally conducted an online survey with seven domain-specific experts to evaluate our taxonomy, archetypes, and decision tree. Table 1 visualizes the research design and highlights the steps, tasks, underlying methods, required and used data, and results.

**Table 1:** Research design.

	Level 1: Taxonomy development	Level 2: Archetype analysis	Level 3: Decision tree development	Level 4: Evaluation of the results
**Steps and tasks**	1.1. Definition of meta-characteristic 1.2. Definition of ending conditions 1.3. Systematic literature review 1.4. 1st iteration (C2E) 1.5. Stepstone analysis and dataset creation 1.6. 2nd - 4th Iteration (E2C)	2.1. Identification of the optimal number of clusters with the elbow and silhouette method 2.2. Cluster analysis 2.3 Derivation of archetypical patterns 2.4 Interpretation of archetypes	3.1. Creation of dataset of the results of Levels 1 and 2 3.2. Dataset split into training and test data with a ratio of 70 %–30 % 3.3. Algorithm running 3.4. Transfer of the results from the algorithm into a decision tree	4.1. Definition of evaluation criteria 4.2. Search for domain-specific experts 4.3. Creation of an online survey 4.4. Conduction of online survey 4.5. Inclusion of survey feedback
**Method/references**	Taxonomy development: Kundisch et al. (2021) and Nickerson et al. (2013) IT PM job advertisements collection: Stepstone.com	Cluster analysis: Kaufman and Rousseeuw (1990), Oberländer et al. (2019) and Saputra et al. (2020)	Decision tree development: Pedregosa et al. (2011)	Evaluation of the results: Sonnenberg and Hevner et al. (2004), Prat et al. (2014), Szopinski et al. (2019) and Sonnenberg and vom Brocke (2012)
**Required/used data**	Academic articles and list of IT PM job advertisements	Classified IT PM job advertisements (taxonomy)	Classified IT PM job advertisements (taxonomy) and corresponding archetypes	Seven domain-specific experts
**Results**	Taxonomy and classification of job advertisements according to our taxonomy's dimensions and characteristics	Archetypes of IT PM job advertisements	Decision support framework for IT PM recruitment	Evaluated results regarding usability, comprehensibility, relevance, completeness, and added value

Level 1 consists of the iterative taxonomy development process. At first, meta-characteristics for our taxonomy are determined, which are decisive and guide the entire taxonomy design; all later characteristics and dimensions for the following classification of the objects are oriented towards them. On the one hand, they describe the topic of our taxonomy; on the other hand, they describe the point of view from which the topic is looked at and the actual idea of our taxonomy. In this study, the meta-characteristics are elements for requirements and benefits for IT PM positions from the job portal Stepstone. Before selecting this portal, we also reviewed others, e.g., Monster, XING, and Indeed. Regarding the hit numbers, detailed requirements, and corporate benefit descriptions, Stepstone provided the most information.

The second step is determining the ending conditions because the taxonomy development process runs in a loop in whose individual iterations the taxonomy is created, extended, adapted, and controlled. At the end of each iteration, it is checked whether all objective and subjective ending conditions proposed by Ref. [26] are fulfilled or whether a further iteration is required. We began our taxonomy development process with the conceptual-to-empirical (C2E) approach to integrate theoretical knowledge from IT project management. For this purpose, we performed a systematic literature review [[55], [56], [57], [58], [59]] based on a keyword-based database search and a subsequently backward, forward, and Google Scholar author and similarity search. This deductive conceptualization identified dimensions and characteristics of the initial taxonomy from relevant literature. The empirical-to-conceptual (E2C) approach was applied in the following iterations, which involved examining 125 job advertisements for IT PM positions [51]. After four iterations, all ending conditions were met, and the final taxonomy was developed (see online Appendix 1).

In level 2, we conducted an archetype analysis to check the applicability of our taxonomy as proposed by Ref. [25]. Therefore, we applied the k-means clustering algorithm, which computes the distances between all objects. It clusters objects into groups to minimize the distances between them and maximize the distances between the clusters themselves [60]. Before executing the clustering analysis, we need to identify the optimal number of clusters (k). The elbow and silhouette methods measure the clusters' cohesion and separation and graphically determine the created clusters’ quality [61]. The elbow method uses the main idea that k-means clustering attempts to minimize the variance. It is a heuristic and the optimal number of clusters can be seen by plotting the variance to the number of clusters. Then the user can see the optimal number by the “elbow” of the curve. The silhouette method is another heuristic that aims to verify how well each point lies in its cluster depending on its size [60]. The elbow and the silhouette method results indicate five clusters as an optimal number for this study.

Based on the identified dimensions, characteristics, and archetypes, we developed a decision tree as a decision support framework for IT PM recruitment in level 3. For the decision tree development, we applied a rule-mining algorithm and followed the guidelines by Ref. [62] via sci-kit learn. This includes the separation of the dataset (classified IT PM job advertisement in accordance with our taxonomy and corresponding archetypes) into training and test data with a ratio of 70 % and 30 %. The identified dimensions and characteristics of our taxonomy are used in the decision tree as the decision classes (questions and answers), the deduced archetypes are the outputs that the decision tree tries to predict. As a result, the decision tree is divided into various questions that must be answered to get a result and suggestions for requirements and receiving corporate benefits according to the five identified archetypes.

In level 4, we evaluated our theoretically and empirically deduced results and findings with target stakeholders. Thus, we evaluated their comprehensibility, relevance, usability, completeness, and added value [54,63,64]. In line with the framework by Ref. [53] we therefore focused on the three questions “who”, “what”, and “how”. We chose practitioners from various industries with domain-specific knowledge and involvement in the IT PM recruitment process with no previous contact with the developed taxonomy, archetypes, and decision support framework as the subject of evaluation (“who”). Regarding the object of evaluation (“what”), we decided on “the recruitment of suitable IT PM” as the real-world problem of investigation. To embed practical knowledge and evaluate our findings' method and content, we decided to perform expert-based online surveys as the method for evaluation (“how”). The evaluation criteria guided the questions. Table 2 gives an overview of the experts’ profiles.

**Table 2:** Experts’ profiles.

Expert ID	Industry	Job description
Exp 1	Communications & technology news service	Consultant for human resources marketing
Exp 2	Vehicle construction/supplier	Recruiter for university graduates
Exp 3	Food and beverage	Human resources specialist
Exp 4	Business consulting	CEO, focus on start-ups and companies in the area of, e.g., human resources
Exp 5	IT and internet	Human resources-IT and people analytics
Exp 6	Human resource services	Human resources consultant
Exp 7	IT services and consulting	CEO

We used qualitative-structured content analysis to deductively analyze the experts’ answers according to the evaluation criteria [65]. The dimensions, characteristics, archetypes, and decision tree were comprehensible for all experts. They further perceived the decision support framework as valuable to identify requirements and corporate benefits depending on the archetype. In addition, all experts regarded our taxonomy and archetypes as valuable in combination with the decision tree and on their own. Nevertheless, the experts had additional ideas and recommendations for improvement. For example, it encompassed the inclusion of salary along with the presentation of requirements and corporate benefits. However, the experts were also aware of a lack of information about salaries in job advertisements. In the first iteration, an initial taxonomy structure following the C2E [26] approach based on the results of a literature review according to Refs. [[55], [56], [57], [58], [59]] was developed. It involved identifying relevant literature by conducting a keyword-based database search in the databases AISeL, IEEE Xplore, SpringerLink, ACM, and JSTOR, with the search string: (“IT project manager” AND “job advertisements” OR “skills” OR “competencies” OR “benefits” OR “selection” OR “recruitment”). This search yielded a total of 45 results. After reviewing the title and abstract and performing a backward, forward, and Google Scholar similarity and author search, we identified 15 articles as relevant for providing a basis for formulating dimensions of the taxonomy. The review of these articles initially revealed ten dimensions (see Table 3). We identified the dimensions D_1_ business knowledge [14,37], D_2_ certificates [39,47], D_3_ soft skills [12,35,41], D_4_ IT skills [12,37], D_5_ communication [11,45,46], D_6_ personal characteristics [11,42,43], D_7_ team management leadership [11,20,46], D_8_ scope management [20], D_9_ project management skills [44], and D_10_ client management [18,45,46].

**Table 3:** Concept matrix of IT PM theory.

Dimension D_*i*_	[11]	[12]	[14]	[18]	[20]	[35]	[37]	[39]	[41]	[42]	[43]	[44]	[45]	[46]	[47]
D_1_ Business Knowledge			x				x								
D_2_ Certificates			x				x	x	x						x
D_3_ Soft Skills		x				x	x		x						
D_4_ IT Skills	x	x				x	x		x						
D_5_ Communication	x			x						x	x		x	x	
D_6_ Personal Characteristics	x									x	x				
D_7_ Team Management and Leadership	x			x	x									x	
D_8_ Scope Management					x										
D_9_ Project Management Skills					x							x			
D_10_ Client Management				x									x		

## 4 Taxonomy development

We applied the E2C approach for the second iteration and selected six IT PM job advertisements drawn from the job portal website Stepstone. Based on the empirical examination of the objects, we eliminated and modified the initial set of dimensions because of insufficient information or violation of the ending conditions. We excluded the dimensions D_1_, D_6_, D_7_, D_8_, and D_10_ because of insufficient information and modified the dimensions D_2_, D_4_, and D_9_. Dimension D_2_ certificates and D_9_ project management skills were merged to the perspective IT PM certificates with the dimensions, respectively the individual certificates: Scaled Agile Framework (SAFe), Project Management Institute (PMI), International Project Management Association (IPMA) level A-D, German association for project management (GPM), Scrum, and Prince2, each with the characteristics compulsory, voluntarily, and not specified. D_4_ IT skills was modified to a perspective with the dimensions Information Technology Infrastructure Library (ITIL), agile working, method or tool expertise, programming skills, and IT architecture, each with the characteristics required and not required. We further added the dimensions of education (characteristics: college, apprenticeship, not specified, advanced education), position (characteristics: junior IT PM, IT PM, senior IT PM), and experience (characteristics: several years, one year, not specified). These dimensions were grouped in the perspective of basic information. We also added the perspective of corporate benefits with the dimensions of home office (characteristics: partly, not specified), childcare allowance, vacation payments, employee events, training opportunities, flexible working hours, company pension plans, job ticket, sports and health activities, and employee discounts (each with the characteristics available, not specified).

Furthermore, we added the dimensions of salary. We added 13 job advertisements in the third iteration in another E2C approach. Soft skills were eliminated as a dimension and modified as a perspective, otherwise, the final conditions were violated. This perspective compromises the dimensions of structured working, assertiveness, communication skills, team-working ability, independent working, goal orientation, sense of responsibility, customer orientation, resilience, and analytical and conceptual mindset, all with the characteristics required and not specified. We further eliminate the dimensions SAFe and salary because of insufficient information. In the previous iteration, not all ending conditions were fulfilled and we thus performed a fourth E2C iteration. We examined 106 further job advertisements. Thereby we neither identified additional nor excluded existing dimensions or characteristics. After this iteration, all ending conditions proposed by Ref. [26] were fulfilled, completing the development process of the taxonomy. The final IT PM position taxonomy comprises 33 dimensions and 77 characteristics (see Table 4). The taxonomy is structured so that the higher-level perspectives, such as basic information or soft skills, are shown in the left-hand column. The dimensions D_1_ to D_33_ are listed in the middle column. Each of the 33 dimensions contains its characteristics in the right-hand column. This means that the D_1_ position can have the characterization junior IT PM, IT PM, or senior IT PM. Only one of the characteristics is allowed to be selected. The brackets behind the characteristics indicate the absolute percentage distribution. This means that 8 % of the 125 job advertisements were looking for junior IT PM, 88 % were looking for IT PM, and 15 % were looking for senior IT PM. Online Appendix 3 shows the entire taxonomy progression.

**Table 4:** Final taxonomy of IT PM job descriptions based on 125 job advertisements including the distributions of the characteristics in the respective dimensions.

Layer 1: Perspective	Layer 2: Dimensions $D_{i}$	Characteristics $C_{i,j}$		
Basic information	$D_{1}$ Position	$C_{1,1}$ Junior IT PM (8 %)	$C_{1,2}$ IT PM (77 %)	$C_{1,3}$ Senior IT PM (15 %)
	$D_{2}$ Experience	$C_{2,1}$ One year (20 %)	$C_{2,2}$ Several years (55 %)	$C_{2,3}$ Not specified (25 %)
	$D_{3}$ Education	$C_{3,1}$ College (39 %)	$C_{3,2}$ Apprenticeship (1 %)	$C_{3,3}$ Not specified (12 %)
		$C_{3,4}$ Advanced education (48 %)		
Soft skills	$D_{4}$ Structured working	$C_{4,1}$ Required (37 %)	$C_{4,2}$ Not specified (63 %)	
	$D_{5}$ Assertiveness	$C_{5,1}$ Required (14 %)	$C_{5,2}$ Not specified (86 %)	
	$D_{6}$ Communication skills	$C_{6,1}$ Required (62 %)	$C_{6,2}$ Not specified (38 %)	
	$D_{7}$ Team-working ability	$C_{7,1}$ Required (38 %)	$C_{7,2}$ Not specified (62 %)	
	$D_{8}$ Independent working	$C_{8,1}$ Required (41 %)	$C_{8,2}$ Not specified (59 %)	
	$D_{9}$ Goal orientation	$C_{9,1}$ Required (10 %)	$C_{9,2}$ Not specified (90 %)	
	$D_{10}$ Sense of responsibility	$C_{10,1}$ Required (25 %)	$C_{10,2}$ Not specified (75 %)	
	$D_{11}$ Customer orientation	$C_{11,1}$ Required (33 %)	$C_{11,2}$ Not specified (67 %)	
	$D_{12}$ Resilience	$C_{12,1}$ Required (6 %)	$C_{12,2}$ Not specified (94 %)	
	$D_{13}$ Analytical/conceptual mindset	$C_{13,1}$ Required (43 %)	$C_{13,2}$ Not specified (57 %)	
IT PM certificates	$D_{14}$ PMI	$C_{14,1}$ Compulsory (1 %)	$C_{14,2}$ Voluntary (15 %)	$C_{14,3}$ Not specified (84 %)
	$D_{15}$ IPMA	$C_{15,1}$ Compulsory (2 %)	$C_{15,2}$ Voluntary (12 %)	$C_{15,3}$ Not specified (86 %)
	$D_{16}$ Scrum master	$C_{16,1}$ Compulsory (3 %)	$C_{16,2}$ Voluntary (13 %)	$C_{16,3}$ Not specified (84 %)
	$D_{17}$ Prince2	$C_{17,1}$ Compulsory (2 %)	$C_{17,2}$ Voluntary (15 %)	$C_{17,3}$ Not specified (83 %)
	$D_{18}$ GPM	$C_{18,1}$ Compulsory (0 %)	$C_{18,2}$ Voluntary (4 %)	$C_{18,3}$ Not specified (96 %)
IT skills	$D_{19}$ ITIL	$C_{19,1}$ Compulsory (1 %)	$C_{19,2}$ Voluntary (10 %)	$C_{19,3}$ Not specified (89 %)
	$D_{20}$ Agile working method	$C_{20,1}$ Required (40 %)	$C_{20,2}$ Not specified (60 %)	
	$D_{21}$ IT architecture knowledge	$C_{21,1}$ Required (30 %)	$C_{21,2}$ Not specified (70 %)	
	$D_{22}$ Method/tool expertise	$C_{22,1}$ Required (74 %)	$C_{22,2}$ Not specified (26 %)	
	$D_{23}$ Programming skills	$C_{23,1}$ Compulsory (6 %)	$C_{23,2}$ Voluntary (9 %)	$C_{23,3}$ Not specified (85 %)
Corporate benefits	$D_{24}$ Home office	$C_{24,1}$ Partly (50 %)	$C_{24,2}$ Not specified (50 %)	
	$D_{25}$ Flexible working hours	$C_{25,1}$ Available (74 %)	$C_{25,2}$ Not specified (26 %)	
	$D_{26}$ Company pension plans	$C_{26,1}$ Available (54 %)	$C_{26,2}$ Not specified (46 %)	
	$D_{27}$ Training opportunities	$C_{27,1}$ Available (62 %)	$C_{27,2}$ Not specified (38 %)	
	$D_{28}$ Employees events	$C_{28,1}$ Available (41 %)	$C_{28,2}$ Not specified (59 %)	
	$D_{29}$ Childcare	$C_{29,1}$ Available (19 %)	$C_{29,2}$ Not specified (81 %)	
	$D_{30}$ Vacation payments	$C_{30,1}$ Available (8 %)	$C_{30,2}$ Not specified (92 %)	
	$D_{31}$ Job ticket	$C_{31,1}$ Available (31 %)	$C_{31,2}$ Not specified (69 %)	
	$D_{32}$ Sport and health activities	$C_{32,1}$ Available (45 %)	$C_{32,2}$ Not specified (55 %)	
	$D_{33}$ Employee discounts	$C_{33,1}$ Available (45 %)	$C_{33,2}$ Not specified (55 %)	

## 5 Cluster analysis and archetype identification

Table 5 shows the cluster analysis results and visualizes the percentage distribution for the identified five archetypes. Each characteristic is color labeled, with 0 % in white and 100 % in dark gray. For example, dimension D_1_ position in Archetype 1 is 83 % at the characteristic C_1,2_ IT PM.

**Table 5:**
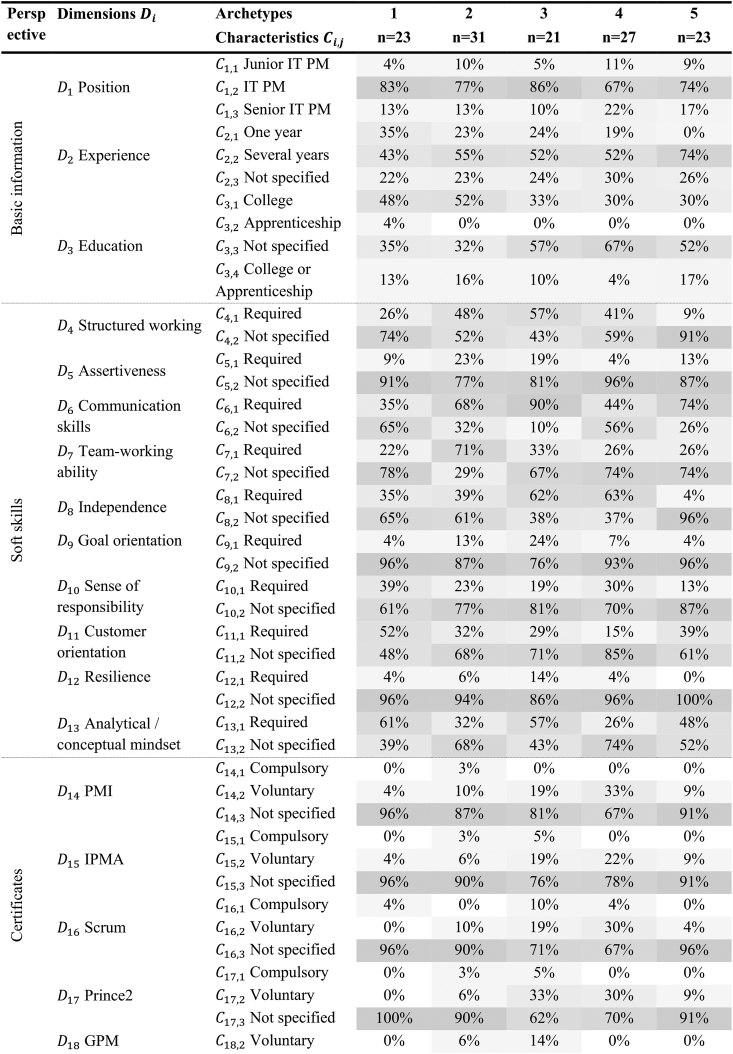

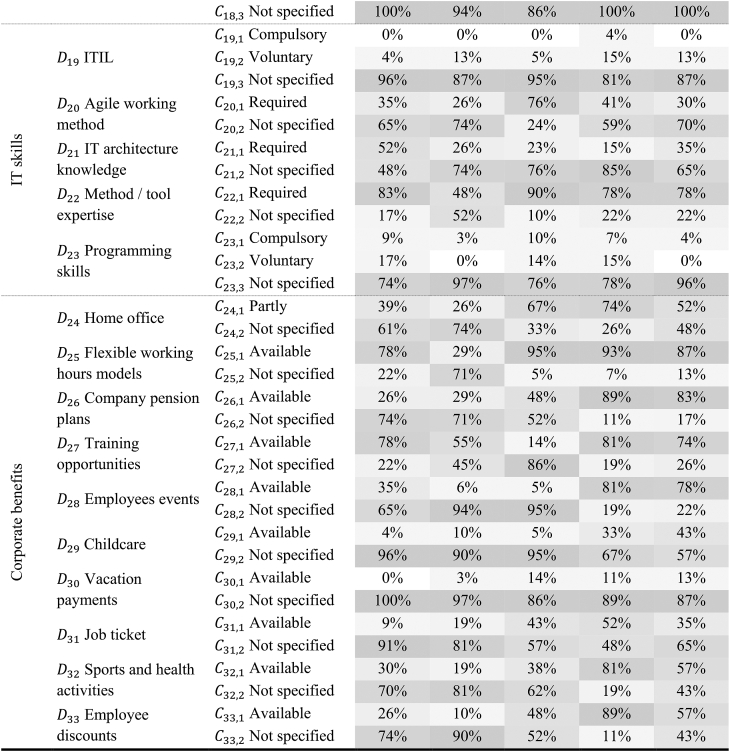
Results of the cluster analysis.

Note: Because of rounding inaccuracies, the sum of columns in a dimension is not always exactly 100 %.

The first archetype includes a wide-ranging focus with normally distributed IT skills and IT PM certifications in contrast to the other archetypes. However, the requirements in terms of IT knowledge, analytical mindset, and programming experience appear higher in this archetype. Furthermore, this archetype requires preferably college-educated employees. Besides, corporate benefits are not advertised frequently. Company pension plans, childcare, job tickets, and vacation payments are offered the least in this archetype. Archetype 2 involves an entry-level environment in which most junior IT PMs are addressed. Here, most college graduates are targeted. Furthermore, this archetype focuses on soft skills. Teamwork, structured work, and assertiveness are in high demand. Moreover, this archetype requires a few IT PM certifications and IT skills. The possibility of working in home office and flexible working hours is offered the least in this archetype, with 26 % and respectively 29 %. Other corporate benefits, such as vacation payments, employee discounts, and company pension plans are scarcely offered. Archetype 3 is PM-focused, with more PM than IT-focused job offers. Furthermore, communication skills are required in 90 % of the job postings and a structured work style in 57 %. In this archetype, many PM certificates are preferably required. These include Prince2, voluntarily, as well as IPMA and GPM. Archetype 4 can be interpreted as human capital investment attraction. Here a variety of skills are required but also many different benefits offered. IT PM certificates such as PMI (33 %), IPMA (22 %), and Scrum (30 %) are needed. In addition, some corporate benefits are offered. Employee discounts, sports and health activities, employee events, training opportunities, and company pension plans are the most frequently mentioned in this archetype. Furthermore, the possibility of working in home office (74 %) and training opportunities (81 %) are offered most often in this archetype. Archetype 5 is experience-oriented. 74 % of the advertisements require several years of experience and especially communication skills are demanded. Overall, job advertisements in this archetype offer the most corporate benefits. This means that over half of the job advertisements promote home office opportunities (52 %), flexible working hours (87 %), company pension plans (83 %), training opportunities (74 %), and employee events (78 %). IT skills and certificates are not required.6. Decision support framework.

**Fig. 1:**
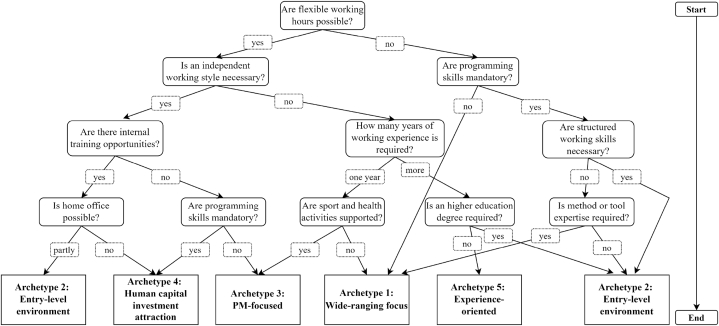
Decision tree for IT PM recruitment.

Based on the identified dimensions, characteristics, and archetypes, we developed a decision tree that serves as a decision support framework for IT PM recruitment (cf. Fig. 1). Identified dimensions from our taxonomy (cf. Table 4) serve as questions and underlying characteristics as answers. By answering four questions, the decision support framework gives archetype-specific suggestions for requirements and benefits for an IT PM (cf. Table 6) corresponding to the identified archetypes (cf. Table 5). Thus, it supports to write target-specific job advertisements to get the attention of the right applicants [24], and assists in conducting interviews and the final selection process. The decision tree is divided into various questions that must be answered to get a recommendation. The reading direction is from top to bottom, starting with the question of flexible working hours and ending with the different archetype recommendations. In the following, we explain the logic and procedure exemplary. The remaining decision tree follows the same logic. The first question relates to whether an organization allows flexible working hours, which can be answered with “yes” or “no”. If flexible working hours are possible, the left side of the decision tree applies. If there are fixed working hours with no flexibility, the right side of the decision tree is pursued.

**Table 6:** Checklist for recommended archetypes.

Archetype	Requirements	Corporate benefits
Archetype 1	*Soft skills*: sense of responsibility, customer orientation, analytical conceptual mindset. *Certificates*: *IT skills*: IT architecture knowledge, method and tool experience, and programming skills.	Training opportunities, flexible working hours.
Archetype 2	*Soft skills*: structured work, assertiveness, communication skills, team working ability. *Certificates*: *IT skills*: agile working method.	Training opportunities.
Archetype 3	*Soft skills*: structured work, communication skills, independent working skills, goal-oriented working, resilience, analytical and conceptual mindset. *Certificates*: PMI, IPMA, Scrum, and Prince2. *IT skills*: agile working method, method and tools expertise.	Flexible working hours, home office, job ticket, employee discounts.
Archetype 4	*Soft skills*: structured work, independent working, responsibility. *Certificates*: Scrum, PMI, IPMA, and Prince2. *IT skills*: ITIL, programming skills, method and tool experience.	Home office, company pension plans, training opportunities, sport and health activities, employee discounts.
Archetype 5	*Soft skills*: communication, customer orientation *Certificates*: *IT skills*: IT architecture knowledge.	Company pension plans, flexible working hours, childcare, employee events, sports and health activities, employee discounts.

To illustrate the decision tree, we describe the outermost branch on the left side as an example. In this case flexible working hours are possible. The next question is whether an independent working style is required. If that is not the case, the next question is about required experience for the IT PM position. However, this branch is not pursued further in this example to reduce the complexity. Instead, we follow the case where an independent working style is required. Then, the next question is about the possibility of internal training opportunities. For the case they are available, the following question is about home office opportunities. If home office is not possible, Archetype 4 is recommended and if it is partly possible, Archetype 2 is recommended. The procedure for all other branches and questions is the same as the described example. All archetype-specific recommended requirements and benefits are shown in Table 6. These archetype recommendations were determined based on the characteristic's percentage distribution in the respective cluster.

## 6 Discussion, theoretical contributions, and practical implications

According to Ref. [12], hard and soft skills are indispensable for a PM to master tasks successfully due to digitalization and an increasingly complex environment [11]. show that the requirements for PM are becoming increasingly extensive; consequently, it is difficult to summarize many competencies into core competencies. To address this gap, we developed a decision support framework for IT PM recruitment using a decision tree based on our taxonomy and specific archetypes. We identified design elements for IT PM recruitment in an iterative process combining scientific literature and real-world IT PM job advertisements.

Examining the distribution of the characteristics in the respective dimensions (see Table 4) shows major differences. Most job advertisements were for IT PM with several years of experience and required advanced education. Regarding soft skills, mostly communication skills, independent working, and an analytical and conceptual mindset were required. The biggest distinctions between the dimensions in the requirements profile are in software skills and certification. Certifications are on a path of increasing popularity [47], but it can be seen that they are not required in many job advertisements. The job advertisements show that employees require just under 30 % of certifications. In job advertisements where certifications are required, certification is often in the context of “ideally”. This shows that most companies that require certifications do not necessarily insist on them but consider certifications a welcome add-on for an applicant. Mostly demanded are PMI, Prince2, IMPA, and Scrum. In addition, although we analyzed IT PM positions, comparatively fewer IT skills were asked. This is consistent with the literature that soft skills are more important than hard skills [13,41]. IT PM should have method and tool expertise and know agile working methods. However, the type and amount of corporate benefits differ among the positions. The most frequently offered corporate benefits are home office, flexible working hours, company pension, and training opportunities, aligning to Ref. [66]. We also identified that job advertisements often mention “attractive compensation” instead of an actual salary. In contrast, according to Ref. [66], providing concrete salary information can increase the organization's attractiveness to applicants.

While our study provides insights into IT PM job offering requirements and benefits using data from Stepstone, many IT PM positions are not advertised in job platforms but rather are filled based on internal promotions or through headhunting which could have a bias on our results. Aligning with [1,41], it is difficult for IT PM to keep their knowledge updated in rapidly changing and evolving environments. In our analysis, we considered published job advertisements. These represent requirements and benefits at a specific time. However, new technologies can lead to requirements that may not yet are considered in current job advertisements. For example, artificial intelligence, cloud computing, or (cyber) security awareness leads to new requirements. The usage of artificial intelligence tools such as ChatGPT, for example, requires good prompt engineering but also the ability to evaluate the results for accuracy. These requirements can be discovered, for example through continuous monitoring of industry trends, cross-functional collaborations, innovation hubs, and participation in industry-specific conferences.

Our taxonomy, archetypes, and decision tree provide three major contributions. First, we developed a theoretically grounded and empirically validated taxonomy of design elements of IT PM job advertisements and identified 33 dimensions and 77 characteristics, classifying 125 IT PM job advertisements. Our taxonomy indicates how IT PM advertisements can be classified, contributing to an empirical and comprehensive knowledge base for researcher. Further, it can be used as a baseline and knowledge base for further theory building (i.e., design theories) [25,27] and as a glossary containing relevant field-specific terminology. In addition, as a second contribution, we provide archetypical patterns of IT PM advertisements and deduce five specific archetypes. By deducing these archetypes, we improve the knowledge of IT PM recruitment. The archetypes show differences and similarities between IT PM advertisements, which a taxonomy alone cannot provide because of its descriptive nature [67]. Further, the patterns enable the identification and further analysis of essential benefits and skills of IT PM and their relations. Along with [25], identifying archetypes also evaluates the developed taxonomy. Researcher can further advance the IT PM recruitment knowledge base based on our results and findings. Third, we contribute to IT PM recruitment decision support providing a decision tree that enables recruitment managers a structured way to identify IT PM requirements fitting for a vacant position. Thus, we show how to expand the taxonomy and archetypes with our decision tree. It also supports recruitment managers in formulating a specific job advertisement suitable for a vacant position. As [11] stated, IT PM competencies change due to its various application fields and the list of IT PM competence requirements become too complex and extensive. Our decision tree supports recruitment managers in identifying which competencies are essential while reducing complexity. A suitable applicant can be found only by identifying the important IT PM competencies for a specific vacant position [24]. This starts before publishing a job advertisement in the development phase of the job advertisement description.

Our evaluation processes showed the applicability and usefulness of our taxonomy, archetypes, and decision tree [53]. In general, all experts perceived our results as beneficial and practically applicable. The evaluation of the results also showed that our taxonomy, archetypes, and decision tree offer a valuable contribution to the everyday work of a recruiter for IT PM positions. Expert 7 particularly emphasizes hard and soft skills, language skills, experience, and mobile working for their positions as important. Additionally, Expert 7 confirmed the usefulness of our taxonomy, archetypes, and decision tree for comparing applicants. Because some companies receive many applicants, our results can support selecting and clustering applicants. Expert 5 said that our decision support framework is valuable to identify which factors shape a potential match between applicants and recruiters or for which types of positions, which dimensions are particularly decisive for the applicants. IT PM job advertisements and applicant reactions can be studied structured and analytically using our taxonomy, archetypes, and decision tree. Furthermore, according to Expert 3 and Expert 4 our decision support framework can make a valuable contribution as a checklist for creating job descriptions and identifying potential matches between applicants and recruiters. Our results can also be used as a benchmark for job interviews. Expert 7 also emphasizes that with a high number of applicants, the decision support framework can be used as a tool to select applicants.

In practice, often many (IT) PM work collaboratively on one project. Our identified archetypes can support the right constellation for these (IT) PM. The archetypes and their corresponding skills enable to compose a group of different (IT) PMs. With the help of our decision tree, it is possible to identify the appropriate archetypes for the respective tasks and skills required and to create one or more job offerings accordingly. A classification according to our archetypes can also take place in job interviews to ensure that the right combination of (IT) PMs is hired for a project implementation. Our taxonomy, archetypes, and decision tree enable researchers and practitioners a consistent knowledge aggregation and understanding of the different dimensions and characteristics of IT PM job advertisements and initiate new research directions. Along with [27,68] our taxonomy and archetypes enable a starting point for theory building and testing by examining the concepts and their relationships. According to Ref. [25], the development process is followed by the results' demonstration, evaluation, and communication. To reduce the complexity of our taxonomy's several dimensions, we developed a decision support framework as a decision tree. This form of visualization fits the purpose of decision support and self-assessment for the target group of IT PM recruiting managers. Our decision tree-based decision support framework enables IT PM recruitment managers a structuring and support on the search for a suitable IT PM applicant. Depending on the answers to the questions, the different archetypes give recruiting managers an overview of frequently used combinations of requirements and corporate benefits. Because of the systematic representation of the combinations between needed requirements and receiving corporate benefits makes it generalizable to other recruitment tasks. The outcomes of the decision support framework support IT PM recruitment managers in the recruitment process of IT PM applicants. The decision tree can help recruiter in the daily business as its services as a checklist for the formulation of job advertisements. Managers and team leaders also can use the decision tree to identify their requirements for the position they are looking for. Moreover, it is also possible to build a decision tree from other angles to enable new opportunities for further decision support. Our results and findings expand the existing knowledge base and contribute to the nascent research field of IT PM recruitment.

## 7 Conclusions, limitations, and further research

With increasing numbers and opportunities for IT PM, we contribute to the knowledge base of IT PM recruitment by developing a decision-support framework for IT PM. We classified 125 IT PM job advertisements and analyzed scientific literature. To structure this complex and dynamic field, we developed a theoretically grounded and empirically validated taxonomy based on the established development process by Refs. [25,26]. Contributing to the descriptive knowledge of IT PM recruitment, our taxonomy classifies IT PM positions according to 33 dimensions and 77 characteristics, structured into the perspectives basic information, soft skills, IT PM certificates, IT skills, and corporate benefits. Based hereon, we derived five IT PM archetypes through a cluster analysis, representing typical characteristic combinations throughout all dimensions. These archetypes classify the current state of IT PM positions into clusters and demonstrate different requirements and corporate benefits for an IT PM. Based on our taxonomy and archetypes, we developed a decision tree-based decision support framework that supports the IT PM recruitment lifecycle, from writing an IT job advertisement to conducting interviews and selecting applicants. Our evaluation with seven recruiters shows that our findings are comprehensive and valuable for the target group.

IT PM in general and especially job advertisements are a dynamic research area. Our research is constrained by limitations. According to Ref. [26], we developed the taxonomy in an extendable way to constantly re-evaluate the results. However, our taxonomy is only a snapshot of the current job market situation. Because of constant changes in the market, employer requirements and demands can change and an updated taxonomy and comparison with new dimensions and characteristics can deliver valuable contributions to research and practice. The second limitation involves the scope of our study. We only looked at job advertisements in Germany on the Stepstone platform. Further research must investigate several countries as well as job advertisement platforms. A web crawling tool can systematically extract and analyze big data of job advertisement elements. This research design can guide the analysis of the cultural impact on IT PM positions, required skills, and receiving corporate benefits and identify differences to increase global generalizability. Third, we did not check the performance of our decision support framework in a real-world recruitment environment. A case study in cooperation with practicing recruiters can go beyond our expert survey. In addition, many positions are not advertised publicly, but are filled internally or through headhunting. We did not consider this in our results and findings. Although we evaluated our results for their completeness with different recruiting experts, further research can analyze this topic in more detail and possibly expand and supplement the scope. For example, interviews with headhunters on this topic can be performed and filled IT PM positions, both through a headhunter and through internal promotions, can be analyzed. This enables a more holistic understanding of the interplay between published job offerings, internal promotions, and headhunting for talent acquisition of IT PM jobs. Further, our study focuses on requirements and benefits for IT PM based on job offerings. It neglects, that many projects operate with multiple IT PM, often arranged in hierarchical structures. Thus, further research can explore how the existence of multiple IT PM influences our results and findings in more detail. In addition, we recognized differences in the advertisement design, i.e., used images and inclusive language while we classified the job advertisements. Further research can address this and identify potential patterns. Nevertheless, our results and findings provide knowledge for researchers and further theory-building and support practitioners in the IT PM recruitment process.

## Availability of data and materials

The datasets used and/or analyzed during the current study are available from the corresponding author on reasonable request.

## CRediT authorship contribution statement

**Christin Karrenbauer:** Writing – review & editing, Writing – original draft, Project administration, Methodology, Formal analysis, Data curation, Conceptualization. **Jana Gerlach:** Writing – review & editing, Writing – original draft, Methodology, Formal analysis, Data curation, Conceptualization. **Michael H. Breitner:** Writing – review & editing, Supervision, Conceptualization.

## Declaration of competing interest

The authors declare that they have no known competing financial interests or personal relationships that could have appeared to influence the work reported in this paper.
