# Supplementary material for: Decision support framework for IT project manager recruitment
Source: Heliyon. 2024 Jan 24;10(3):e24685. doi: 10.1016/j.heliyon.2024.e24685 (PMC10850423; doi:10.1016/j.heliyon.2024.e24685)

Decision support framework for IT project manager recruitment

**Appendix 1. Objective and subjective ending conditions.**

| **Objective ending conditions** | **1^st^ I** | **2^nd^ I** | **3^rd^ I** | **4^th^ I** |
| --- | --- | --- | --- | --- |
| All objects or a representative sample of objects have been examined. |  | (6) ● | (13) ● | (106) ● |
| No object was merged with a similar object or split. |  |  |  | ● |
| At least one object is classified under every characteristic. |  |  | ● | ● |
| No new dimensions or characteristics were added in the last iteration. |  |  |  | ● |
| No dimensions or characteristics were merged or split in the last iteration. |  |  |  | ● |
| Every dimension is unique and not repeated. | ● | ● | ● | ● |
| Every characteristic is unique within its dimension. | ● | ● | ● | ● |
| Each cell is unique and is not repeated. | ● | ● | ● | ● |
| **Subjective ending conditions** | | | | |
| Concise: Dimensions and characteristics are limited. |  |  |  | ● |
| Robust: Sufficient number of dimensions. |  |  | ● | ● |
| Comprehensive: Identification of all relevant dimensions & characteristics. |  | ● | ● | ● |
| Extendable: Possibility to easily add dimensions and characteristics in the future. |  |  | ● | ● |
| Explanatory: Dimensions and characteristics sufficiently explain the objects. |  |  |  | ● |

**Appendix 2. Findings of the first C2E iteration.**

| **Dimension D*_i_*** | Alvarenga et al. (2020) | Ashan et al. (2013) | Asplund (2006) | Crawford (2005) | Dillon et al. (2011) | Green (1989) | Jiang et al. (1998) | Keil et al. (2013) | Millhollan and Kaarst-Brown (2016) | Napier et al. (2009) | Ribeiro et al. (2021) | Sampaio et al. (2021) | Skulmoski & Hartman (2010) | Soroka-Potrzebna (2021) | Stevenson & Starkweather (2010) |
| --- | --- | --- | --- | --- | --- | --- | --- | --- | --- | --- | --- | --- | --- | --- | --- |
| D_1_ Business Knowledge |  | x | x |  |  |  |  |  |  |  |  |  |  |  |  |
| D_2_ Certificates |  | x | x |  |  |  |  |  | x |  |  |  |  | x | x |
| D_3_ Soft Skills |  |  | x | x |  |  |  |  |  |  | x |  |  |  | x |
| D_4_ IT Skills | x |  | x | x |  |  |  |  |  |  | x |  |  |  | x |
| D_5_ Communication | x |  |  |  | x | x | x |  |  | x |  | x |  |  |  |
| D_6_ Personal Characteristics | x |  |  |  |  | x | x |  |  |  |  |  |  |  |  |
| D_7_ Team Management and Leadership | x |  |  |  |  |  |  | x |  | x |  | x |  |  |  |
| D_8_ Scope Management |  |  |  |  |  |  |  | x |  |  |  |  |  |  |  |
| D_9_ Project Management Skills |  |  |  |  |  |  |  | x |  |  |  |  | x |  |  |
| D_10_ Client Management |  |  |  |  | x |  |  |  |  | x |  |  |  |  |  |

**Appendix 3. Summary of taxonomy progression and dimensions**


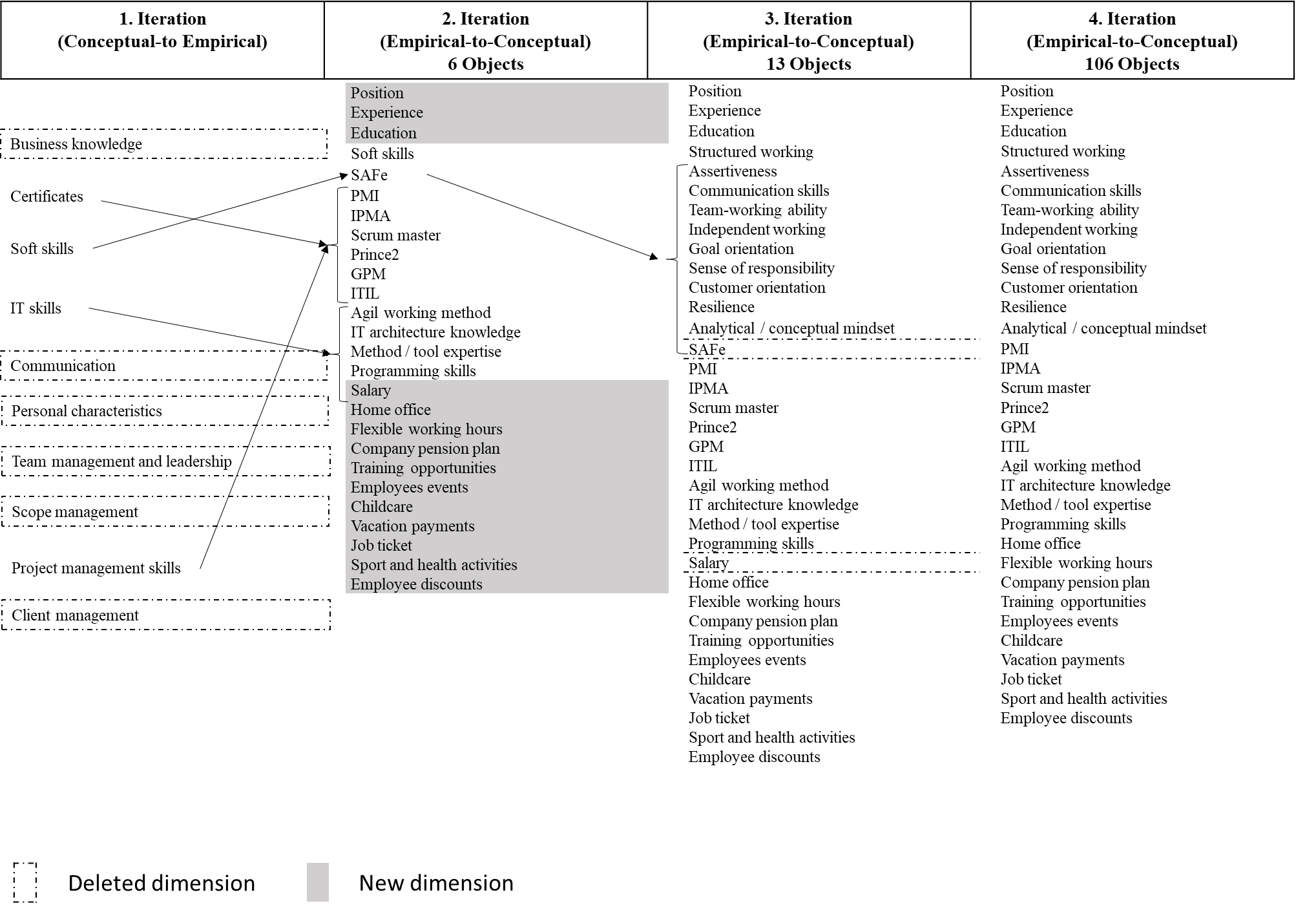

Supplement: Multimedia component 1 [file mmc1.docx]
